# Rapid genomic analysis for early identification of complement abnormalities in adults with transplant-associated thrombotic microangiopathy

**DOI:** 10.1016/j.htct.2026.106497

**Published:** 2026-07-30

**Authors:** Jacopo Mariotti, Stefania Bramanti, Luigi Porcaro, Gianluigi Ardissino, Maria Teresa Pagliari, Silvia Spena, Andrea Cairo, Samantha Griffini, Barbara Sarina, Daniele Marchelli, Eloisa Arbustini, Mario Urtis, Antonio Tescari, Armando Santoro, Flora Peyvandi, Massimo Cugno

**Affiliations:** a Department of Oncology/Hematology, IRCCS Humanitas Research Hospital, Rozzano, Milan, Italy; b Hematology and Stem Cell Transplantation Division, Hospital Legnano, Legnano, Italy; c Medical Genetics Laboratory, Fondazione IRCCS Ca' Granda Ospedale Maggiore Policlinico, Milan, Italy; d Center for HUS Prevention, Control and Management at Pediatric Nephrology, Dialysis and Transplant Unit, Fondazione IRCCS Ca' Granda Ospedale Maggiore Policlinico, Milan, Italy; e Department of Internal Medicine, SC Medicina-Emostasi e Trombosi, Fondazione IRCCS Ca' Granda Ospedale Maggiore Policlinico, Milan, Italy; f Department of Pathophysiology and Transplantation, Università degli Studi di Milano, Milan, Italy; g Centre for Inherited Cardiovascular Diseases, Scientific Department, Fondazione IRCCS Policlinico San Matteo, Pavia, Italy; h Department of Biomedical Sciences, Humanitas University, Pieve Emanuele, Milan, Italy

**Keywords:** Transplant-associated thrombotic microangiopathy, Allogeneic hematopoietic cell transplantation, Complement, Sc5b-9, Nanopore sequencing

## Abstract

**Background:**

Transplant-associated thrombotic microangiopathy is a severe and often fatal complication of allogeneic hematopoietic cell transplantation; early identification of the involved mechanisms may enable timely therapy.

**Study design:**

Adult recipients of allogeneic hematopoietic cell transplantation (n = 195) were studied in the early post-transplant period. The recent harmonizing criteria from the world's leading blood and marrow transplant societies, such as ≥4 of 7 specific clinical/laboratory features for thrombotic microangiopathy diagnosis were applied. Plasma levels of sC5b-9, a marker of complement activation, was measured by an enzyme-linked immunosorbent assay and complement-related genes in blood cells were evaluated by both rapid genomic analyses using nanopore sequencing and conventional methods (targeted next generation sequencing and multiplex ligation-dependent probe amplification assay).

**Results:**

Ten patients who met ≥4 criteria (confirmed transplant-associated thrombotic microangiopathy) had high 1-year non-relapse mortality (60%) and high complement activation. In this group, plasma levels of sC5b-9 were higher than in patients without confirmed disease (p-value = 0.04) and normal controls (p-value <0.001). At the same time (after full chimerism), they showed seven rare variants of complement related genes (minor allele frequency <0.03). Rapid genomic analysis identified these alterations with complete concordance to conventional methods.

**Conclusions:**

These results support the utility of applying harmonized criteria for early diagnosis of transplant-associated thrombotic microangiopathy and performing rapid genomic analysis with nanopore sequencing for the identification of variants in complement-related genes within 72h. Future studies on larger cohorts could explore the integration of rapid genomic analysis in this patient population and potentially for screening donors whose cells may carry genetic alterations for real-time clinical decision making.

## Introduction

Transplant-associated thrombotic microangiopathy (TA-TMA) is a rare but often fatal complication of allogeneic hematopoietic stem cell transplantation (allo-HSCT). Characterized by hemolysis and platelet consumption, this condition often targets the kidneys, leading to significant organ dysfunction and hypertension [1]. TA-TMA can involve other organs, including the intestine, brain, lungs, skin, liver, and testes [2]. The prevalence of TA-TMA ranges from as low as 3% to as high as 39% of transplants with the related fatality rate having been reported in up to 84% of cases [3, 4, 5]. The variability in prevalence reflects the heterogeneity of diagnostic criteria employed in the different hematology centers.

Although histological confirmation remains the gold standard for diagnosis, bleeding risks often preclude biopsy, prompting the development of clinical diagnostic criteria [6, 7, 8, 9, 10, 11]. TA-TMA typically occurs in the early post-transplant period (usually within 100 days), a time when patients are exposed to other causes of thrombocytopenia, anemia and organ damage such as infections, immune-mediated injury and drug toxicity [12]. The identification of TA-TMA biomarkers has focused on the underlying pathophysiology, particularly a widely accepted three-hit model: (1) pre-existing endothelial injury or complement activation susceptibility; (2) toxicity from the conditioning regimen; and (3) post-transplant triggers, such as medications, alloreactivity, or infections [13]. In the last few years, hyperactivation of the complement system has been considered in the pathogenesis of TA-TMA, with a focus on a possible genetic predisposition leading to abnormal activation of the complement cascade [14, 15, 16]. Abnormalities in complement-related genes can also be transferred from the donor to the recipient after full bone marrow chimerism [17].

TA-TMA continues to be a difficult diagnosis, in part due to the lack of universally accepted criteria and the overlapping features with other post-transplant complications, including sinusoidal obstructive syndrome (SOS), veno-occlusive disease (VOD) and graft-versus-host disease (GvHD) [18]. To harmonize diagnostic approaches, the European Society for Blood and Marrow Transplantation, the American Society for Transplantation and Cellular Therapy, the Asia-Pacific Blood and Marrow Transplantation Group, and the Center for International Blood and Marrow Transplant Research (EAABMT) recently endorsed a consensus definition [2]. Based on the Jodele criteria initially developed for pediatric population [11], TA-TMA is diagnosed when four of seven key features occur twice within 14 days: anemia, thrombocytopenia, hypertension, elevated LDH, schistocytes, proteinuria, and elevated soluble C5b-9. These criteria facilitate early diagnosis of TA-TMA; however, the early identification of mechanisms involved in the pathogenesis of the disease is essential for the choice of a timely therapy.

This study applied the EAABMT criteria to adult patients with suspected post-allo-HSCT TA-TMA to identify those at high risk of mortality and to evaluate the prevalence of complement system genetic abnormalities. The variations in genes encoding proteins involved in the activation and control of the complement system were evaluated both with traditional methods (next-generation sequencing and multiplex ligation–dependent probe amplification) [19,20] and with rapid genomic analysis with nanopore sequencing [21, 22, 23], an approach that has been used in other forms of thrombotic microangiopathies [22,23] that achieves diagnoses in <72h [23].

## Patients and methods

### Patients

Between January 1st 2018 and September 31st 2021, 195 consecutive adult patients received an Allo-HSCT at the Humanitas Cancer Center (Rozzano, Italy) for hematologic malignancies. All adult patients with hematologic malignancies undergoing their first allo-HSCT from any graft source (blood marrow or peripheral blood stem cells) and after any type of conditioning regimen were included. The hematopoietic cell transplantation comorbidity index (HSCT-CI) was calculated according to Sorror et al. [24]. Patients were routinely monitored for complete blood counts, LDH, and urine protein levels at least once a week. Blood pressure was monitored daily during hospital admission and on every outpatient visit. Screening features for TA-TMA comprised the seven EAABMT criteria: anemia, thrombocytopenia, elevated LDH, hypertension, schistocytes, proteinuria and sC5b-9 plasma levels (marker of complement activation). When at least three of these characteristics occurred simultaneously or remained unexplained over time, further investigations were carried out, and TA-TMA was diagnosed when at least four of the seven EAABMT features occurred twice within 14 days, as recommended by Schoettler et al. [2]. A total of sixteen patients were suspected of TA-TMA. Their demographic characteristics are summarized in Table 1. *ADAMTS13* (a disintegrin and metalloproteinase with a thrombospondin type 1 motif, member 13), soluble C5b-9 (sC5b-9) complex and complement genetic studies were evaluated for blood samples obtained at the time of TA-TMA suspicion.

**Table 1:** Demographic and clinical characteristics of patients subjected to allogeneic hematopoietic stem cell transplantation with suspected transplant-associated thrombotic microangiopathy (TA-TMA).

Pts	Age	Disease Type	Disease Status	# Previous line therapy	Donor Type	D/R Sex	D/R CMV	D/R Blood group	HSCT-CI	Graft Source	Conditioning Regimen	GvHD Prophylaxis
1	47	CMML	Active	None	Haplo	F→M	Pos/Pos	A+/A+	0	PBSC	TBF MAC	PTCy
2	53	HL	CR	6	UD (7/8)	MM	Pos/Neg	0+/0+	2	PBSC	TBF RIC	PTCy
3	35	ALL-T	CR	1	MRD	M→F	Neg/Pos	0-/A+	3	BM	TBF MAC	ATG
4	35	ALL-T	CR	1	Haplo	F→M	Pos/Pos	A+/A+	2	PBSC	TBF MAC	ATG
5	46	HL	CR	5	Haplo	F→M	Pos/Pos	A+/A-	0	PBSC	Baltimora	PTCy
6	25	AML	CR	4	CB	F→M	Neg/Pos	0+/0+	3	CB	TBF RIC	ATG
7	68	HL	CR	4	Haplo	M→F	Neg/Neg	0+/A+	4	PBSC	Baltimora	PTCy
8	64	MDS	Active	1	Haplo	MM	Pos/Pos	B+/A+	6	PBSC	TBF RIC	PTCy
9	68	AML	CR	2	Haplo	M→F	Pos/Pos	A+/0+	3	PSC	TBF RIC	PTCy
10	67	MDS	CR	1	MUD	F→M	Pos/Pos	A+/A+	0	PBSC	TBF RIC	ATG
11	68	MDS	CR	1	Haplo	MM	Neg/Neg	A+/0+	2	PBSC	TBF RIC	PTCy
12	40	ALL Ph+	CR	1	MUD	MM	Pos/Pos	A+/B+	2	PBSC	TBF MAC	ATG
13	69	MCL	CR	5	Haplo	MM	Neg/Pos	0+/0+	0	PBSC	Baltimora	PTCy
14	65	HL	CR	4	MRD	MM	Pos/Pos	A+/0+	4	PBSC	Baltimora	PTCy
15	46	MDS	Active	None	MUD	F→F	Pos/Pos	0+/0+	3	PBSC	TBF RIC	ATG
16	56	FCL	CR	6	MRD	M→F	Pos/Neg	AB+/0+	6	PBSC	Baltimora	PTCy

ALL: acute lymphoblastic leukemia; AML: acute myeloid leukemia; ATG: anti-thymoglobulin; CB: cord blood; CMML: chronic myelomonocytic leukemia; CMV: cytomegalovirus; CR: complete remission; D/R: donor/recipient; F→M: female→male; FL: follicular lymphoma; GvHD: graft versus host disease; Haplo: haploidentical; HSCT-CI: hematopoietic cell transplantation comorbidity index; HL: Hodgkin lymphoma; MAC: myeloablative conditioning regimen; MCL: mantle cell lymphoma; MDS: myelodysplastic syndrome; MRD: matched related donor; MUD: matched unrelated donor; PBSC: peripheral blood stem cell; pH+: Philadelphia positive; PTCy: post-transplant cyclophosphamide; RIC: reduced intensity conditioning; TA-TMA: transplant-associated thrombotic microangiopathy; TBF: thiotepa-busulfan-fludarabine; UD: unrelated donor.

The study was approved by the Lombardia 5 Ethics Committee (no. TAM ONC/OSS-02/2025). All patients gave their written informed consent and all procedures were performed in line with the Helsinki Declaration and the code of good clinical practice.

## Methods

### Soluble C5b-9 measurement

Blood samples were collected from an antecubital vein by clean venipuncture with minimal stasis using a 21 G butterfly needle in tubes containing ethylenediaminetetraacetic acid (EDTA: final concentration 10 mM). The samples were centrifuged within one hour for ten minutes at 2000 g at room temperature. The plasma was removed, divided into aliquots and stored at −80 °C until tested. Plasma levels of soluble C5b-9 (sC5b-9) were measured using a solid-phase assay (MicroVue Complement SC5b-9 Plus EIA kit, Quidel Corporation, San Diego, CA, USA) whose intra- and inter-assay coefficients of variation were respectively 6.8% and 13.1%; the lowest detection limit was 3.7 ng/mL. For sC5b-9 measurements, twenty healthy subjects (ten women and ten men; median age: 48 years; range: 20–70 years) served as normal controls.

### Anti-factor H antibody assay

Anti-factor H autoantibodies were determined by ELISA, using purified factor H for capture and anti-human IgM, IgG, and IgA antibodies for detection [18]. Purified factor H (Calbiochem, EMD Chemicals, San Diego, CA, USA; 10 mg/mL in phosphate-buffered saline at pH 7.4) was coated overnight onto microtitration plates, and, after washing to avoid non-specific binding, the wells were coated with bovine serum albumin. After additional washes, 1:20 diluted serum samples were added and incubated at room temperature for 45 min. After further washing, the factor H-bound immunoglobulins were detected by class-specific mouse monoclonal anti-IgM, -IgG, or -IgA antibodies (Sigma Aldrich, St Louis, MO, USA). Peroxidase-conjugated anti-mouse antibodies (Sigma-Aldrich), developed with ortho-phenylenediamine, were used for detection. Optical density (OD) was measured at 490 nm. Results were expressed in units per mL (U/mL) with reference to an internal standard (serum from a patient with a high anti-factor H antibody titer) arbitrarily fixed at 100 U/mL. In order to avoid the confounding effect of natural antibodies, it was decided to use the maximum level observed in normal subjects as the cut-off between normal and abnormal levels.

### ADAMTS13 activity

*ADAMTS13* activity plasma levels were measured with a fluorescence resonance energy transfer assay that uses a synthetic 73-amino acid peptide (FRETS-VWF73), as previously described [25]. The intra- and inter-assay coefficients of variation were 6% and 9.5%, respectively.

### DNA extraction, quantity and quality control

Genomic DNA was isolated from blood samples using the QIAsymphonySP automated platform (Qiagen GmbH, Hilden, Germany). DNA quality was assessed using the NanoDrop™ 2000c spectrophotometer (ThermoFisher, Waltham, MA, USA) while quantification was performed using the Qubit dsDNA HS assay kit (Thermo Fisher) and the Qubit Fluorimeter (Thermo Fisher). For nanopore sequencing, DNA integrity was also evaluated using the ScreenTape Genomic DNA kit (Agilent Technologies, Santa Clara, CA, USA) and the 4150 TapeStation System (Agilent Technologies). All measurements were carried out following manufacturer instructions.

### Targeted next generation sequencing (NGS)

Nucleotide variations were detected by NGS on the MiSeq platform (Illumina) using the ‘targeted sequencing’ technique (SureSelect XT HS RevB ILM – Tier1 Target Enrichment System, Agilent Technologies) employing a multiple gene custom panel comprising *CFH* (NM_000186.4), *MCP/CD46* (NM_002389.4), *CFI* (NM_000204.5), *C3* (NM_000064.4), *CFB* (NM_001710.6), *THBD* (NM_000361.3), *DGKE* (NM_003647.3), *CFHR1* (NM_002113.3), *CFHR3* (NM_021023.6), *CFHR5* (NM_030787.4), with average depth in target regions greater than 100×. To identify putative causative variants, NGS data were processed and filtered using the Agilent Alissa suite, specifically the Align & Call and Interpret modules (Agilent Technologies). Variants with a minor allele frequency (MAF) <3% (1000 Genome Phase 3 and gnomAD databases) were selected. All variants identified by NGS analysis were then confirmed by the standard Sanger sequencing method. The potential impact of amino acid changes was assessed by *in silico* analysis (VarSome [https://varsome.com], Franklin by genoox [https://franklin.genoox.com/]) to predict the functional significance of unpublished or uncommon variants. Variant frequencies were also cross-referenced against the ExAC database (Exome Aggregation Consortium, [http://exac.broadinstitute.org]) to evaluate their prevalence in the general population. The genetic variant classification of the database of complement genetic variants (http://www.complement-db.org/home.php) was also considered [19,20].

### Multiplex ligation-dependent probe amplification assay (MLPA)

Multiplex ligation-dependent probe amplification assay (MLPA kit P236, MRC—Holland, Amsterdam, The Netherlands) was used to identify *CFHR3*/*CFHR1* copy number and macro rearrangements such as *CFH*/*CFH*-Related hybrid genes. Raw data were analyzed by Coffalyser.net (https://www.mlpa.com) and the relative dosage ratio was calculated [19,20].

### Nanopore sequencing

Prior to library preparation, 3 µg of genomic DNA was sheared using g-Tubes (Covaris, Woburn, MA, USA) by centrifugation at 3500 x g for 1 min at room temperature, yielding 10-kb fragments. Library preparation was carried out using the Ligation Sequencing Kit SQK-LSK114 (Oxford Nanopore Technologies, Oxford, UK). The manufacturer protocol was modified slightly by increasing the incubation times of the DNA repair, end prepare, and ligation steps.

PromethION flow cells (FLO-PRO114; Oxford Nanopore Technologies) were loaded on a PromethION 24 instrument. Following flow cell priming, 50 fmol of library was loaded per flow cell according to the manufacturer’s instructions. DNA sequencing, data acquisition, and real-time basecalling were performed for 72h using MinKNOW software with default settings.

Data analysis was initially performed using the EPI2ME software (Oxford Nanopore Tecnologies) installed on the PromethION 24 instrument, using default settings. The wf-human variation pipeline was used to simultaneously call Single Nucleotide Polymorphisms (SNP), structural variants, Copy Number Variants (CNV) and Short Tandem Repeats (STR) using the CRCh38 as reference genome.

Then, data were blindly submitted to another center for independent analysis using a different pipeline. In this second pipeline, the sequencing data were aligned against the GRCh38 reference with Minimap2 [26] and the alignments were analyzed with CNVKit [27], a software designed to identify CNV. The CNVKit is primarily focused on hybrid capture sequencing data. Although a default analysis pipeline is provided by the developers, the tool execution was customized to better cope with the analyzed data. The workflow included:

i. Preparation of the target region with the *target –split* command (default value of 267 was used for *–average-size* parameter).
ii. Use of a blank anti-target for both reference definition and sample analysis.
iii. Definition of the reference copy number from the alignment profiles of the wt samples selected by a visual inspection carried out with Integrative Genomics Viewer (IGV) of the CHFR1–5 locus [28]
iv. Sample analysis with the serial execution of *coverage, fix, segment (*using the *Hmm-germline* method*)* and *call* (using custom thresholds for copy number calling: −1.1, −0.6, 0.4, 0.7) commands.

### Statistical analysis

Due to a non-normal distribution of data according to the Kolmogorov-Smirnov test, quantitative variables are expressed as median, ranges (minimum–maximum) and/or interquartile range (IQR). Categorical variables are expressed as an absolute number. The nonparametric Mann-Whitney U test was used to compare different groups. Statistical significance was defined as a p-value <0.05. Outcomes were defined according to the European Society for Blood and Marrow Transplantation (EBMT) statistical guidelines. Cumulative incidence estimates were used to account for competing risks in the study population. Differences between cumulative incidence curves in the presence of relapse as competing risk, were tested using the Gray method [29]. Results are presented as 1-year non-relapse mortality. Statistical analyses were performed using SAS software version 9.4 (SAS Institute Inc, Cary, NC, USA) and IBM Statistical Package for Social Sciences (SPSS) version 29.0 (IBM Corp, Armonk, NY, USA). The impact of TA-TMA on overall survival was analyzed employing the Cox proportional hazard model where TA-TMA was evaluated as the time dependent variable (STATA version 14.2, StataCorp, TX, USA).

## Results

### Transplant-associated thrombotic microangiopathy diagnosis

**Figure 1:**
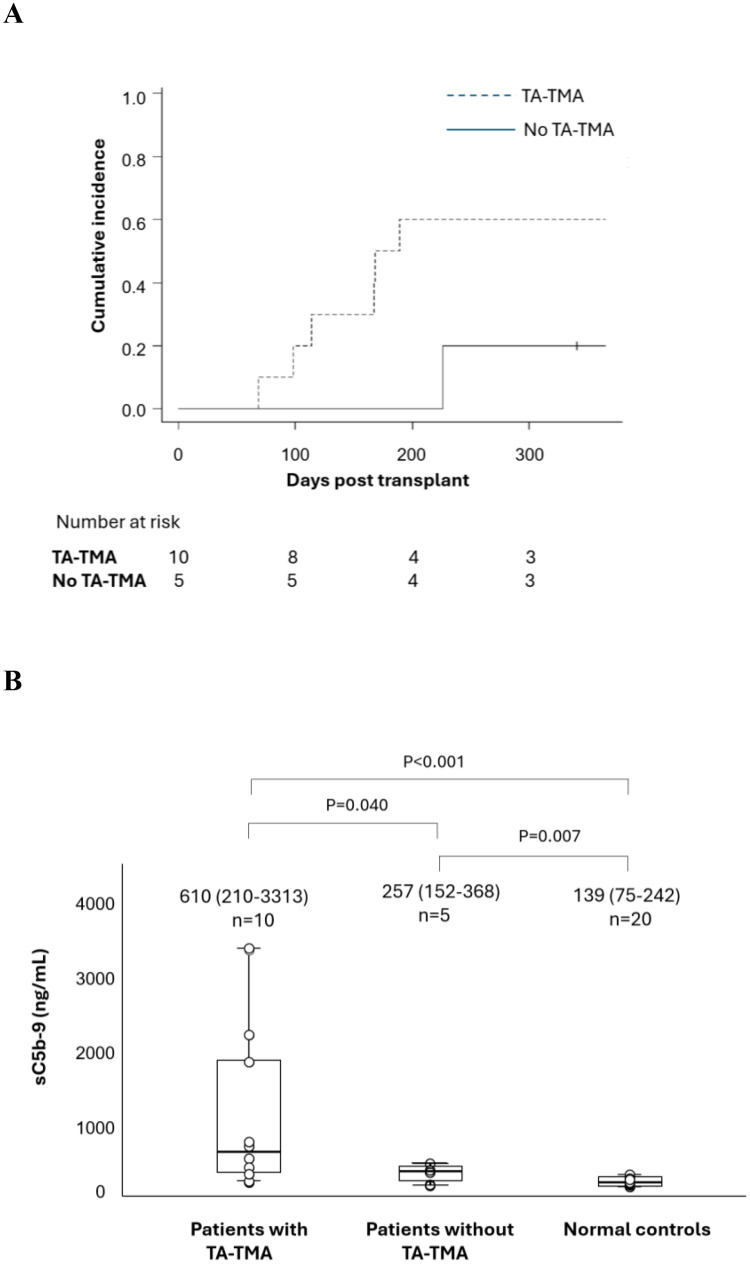
**A**. Incidence of non-relapse mortality in transplant-associated thrombotic microangiopathy (TA-TMA). The figure shows the cumulative incidence of 1-year non-relapse mortality of 15 patients with suspected diagnosis of TA-TMA that was confirmed in ten (TA-TMA) and not confirmed in five (No TA-TMA). **B**. Plasma levels of soluble C5b-9 (sC5b-9) in TA-TMA. The figure shows plasma levels of sC5b-9 in ten patients with TA-TMA, five patients without TA-TMA and 20 healthy subjects that served as controls. Boxes show median value and interquartile range while whiskers show 95% confidence interval.

Of the 16 patients with suspected TA-TMA (Table 1), one (Patient #1) presented *ADAMTS13* levels <3%. Consequently, a diagnosis of thrombotic thrombocytopenic purpura (TTP) was made, and the patient was excluded from the study. The remaining 15 patients (Table 2) comprised the cohort with suspected TA-TMA. The median time to suspected diagnosis was 80 days post-transplant (IQR: 57–95 days). For 13 of these patients, TA-TMA was suspected within the first 100 days post-transplant, while the remaining two were identified at a later stage (170 and 220 days) with both the latter having concurrent chronic graft-versus-host disease (GvHD). Concomitant post-transplant complications occurred in all patients; either acute or chronic GvHD in 11, viral infections in seven and probable invasive fungal disease in one (Table 2). More details are reported in Table 3. Of the 15 patients, five had <4 EAABMT criteria, ruling out TA-TMA; one of them died because of disease relapse and one for acute GvHD. Ten patients had ≥4 EAABMT criteria and were diagnosed with TA-TMA: four criteria in five patients and >4 in the other five (Table 2). Table 4 shows the values of hemoglobin, platelets, lactate dehydrogenase, Coombs test, blood pressure, prothrombin time and activated thromboplastin time. Six of these ten patients were switched from cyclosporine to sirolimus with four showing clinical improvement, one of whom also received eculizumab treatment (Table 3). At 1-year post-transplant, seven of the ten patients had died, one because of disease relapse and six due to post-transplant side effects (Table 3). The cumulative incidence of 1-year non-relapse mortality was 20% in patients with <4 EAABMT criteria and 60% in patients with ≥4 EAABMT criteria (Figure 1A) (p-value = 0.07). Overall survival was further compared between the cohort of ten patients with confirmed diagnosis of TA-TMA and 185 subjects without TA-TMA using the Cox proportional hazard model where TA-TMA was analyzed as the time dependent variable: patients developing TA-TMA had five-fold increased risk of death compared with those without TA-TMA (hazard ratio [HR]: 5.19; 95% confidence incidence: 2.45–10.98; p-value <0.001) Table 5

**Table 2:** Clinical and laboratory characteristics of patients with suspected transplant-associated thrombotic microangiopathy (TA-TMA) after exclusion of the patient with thrombotic thrombocytopenic purpura.

aGvHD: acute graft versus host disease; Allo-HSCT: allogeneic hematopoietic stem cell transplantation; BKV: polyomavirus BK; CMV: cytomegalovirus; cGvHD: chronic graft versus host disease; EAABMT criteria: criteria from the consensus of the world’s leading societies for Blood and Marrow Transplant Research; G2/G3: grade 2 or 3 aGvHD; HHV6: herpes virus 6; IFD: invasive fungal disease.

Patients with confirmed diagnosis of transplant-associated thrombotic microangiopathy (TA-TMA) are highlighted in grey.

**Table 3:** Timing of graft-versus-host disease, veno-occlusive disease (VOD), infections and initiation of immunosuppressive therapy from the diagnosis of transplant-associated thrombotic microangiopathy (TA-TMA).

Patients with confirmed diagnosis of transplant-associated thrombotic microangiopathy (TA-TMA) are highlighted in grey. The minus sign refers to the days before the diagnosis of TA-TMA and the plus sign refers to the days after the diagnosis of TA-TMA.

aGvHD: acute GvHD; BP: blood pressure; BSI: blood stream infection; cGvHD: chronic GVHD; Hb: hemoglobin; LDH: lactate dehydrogenase; NA: not available; PLT: platelet; SARS-CoV2: severe acute respiratory syndrome coronavirus 2.

**Table 4:** Laboratory and clinical characteristics of patients with transplant-associated thrombotic microangiopathy (TA-TMA).

Hb: hemoglobin; PLT: platelet; LDH: lactate dehydrogenase; DAT: direct antiglobulin test (direct Coombs test); NV: normal values; PT/aPTT: prothrombin time/activated thromboplastin time.

Patients with confirmed diagnosis of transplant-associated thrombotic microangiopathy (TA-TMA) are highlighted in grey.

**Table 5:** Chimerism and supplementary donor characteristics.

CB: Cord blood; Haplo: Haploidentical transplant; MRD: Matched related sibling; MUD: Matched unrelated donor; NA: not available; UD: Unrelated donor (≥7/8 disparity).

Patients with confirmed diagnosis of transplant-associated thrombotic microangiopathy (TA-TMA) (EAABMT criteria ≥4) are highlighted in grey.

Consistent with TA-TMA diagnosis (≥4 EAABMT criteria), the ten patients with TA-TMA had sC5b-9 plasma levels (median 610 ng/mL; range 210–3313 ng/mL) higher than the five patients with <4 EAABMT criteria (257 ng/mL; 152–368 ng/mL) (Figure 1B; p-value = 0.040). In patients with TA-TMA, sC5b-9 plasma levels were also higher than those of 20 healthy controls (131 ng/mL; 75–242 ng/mL) (p-value <0.001). None of the patients in this cohort tested positive for anti-factor H autoantibodies (IgG, IgM, or IgA).

### Genetic results

At the time of genetic testing, full chimerism was evident in all 15 patients (Table 5).

**Figure 2:**
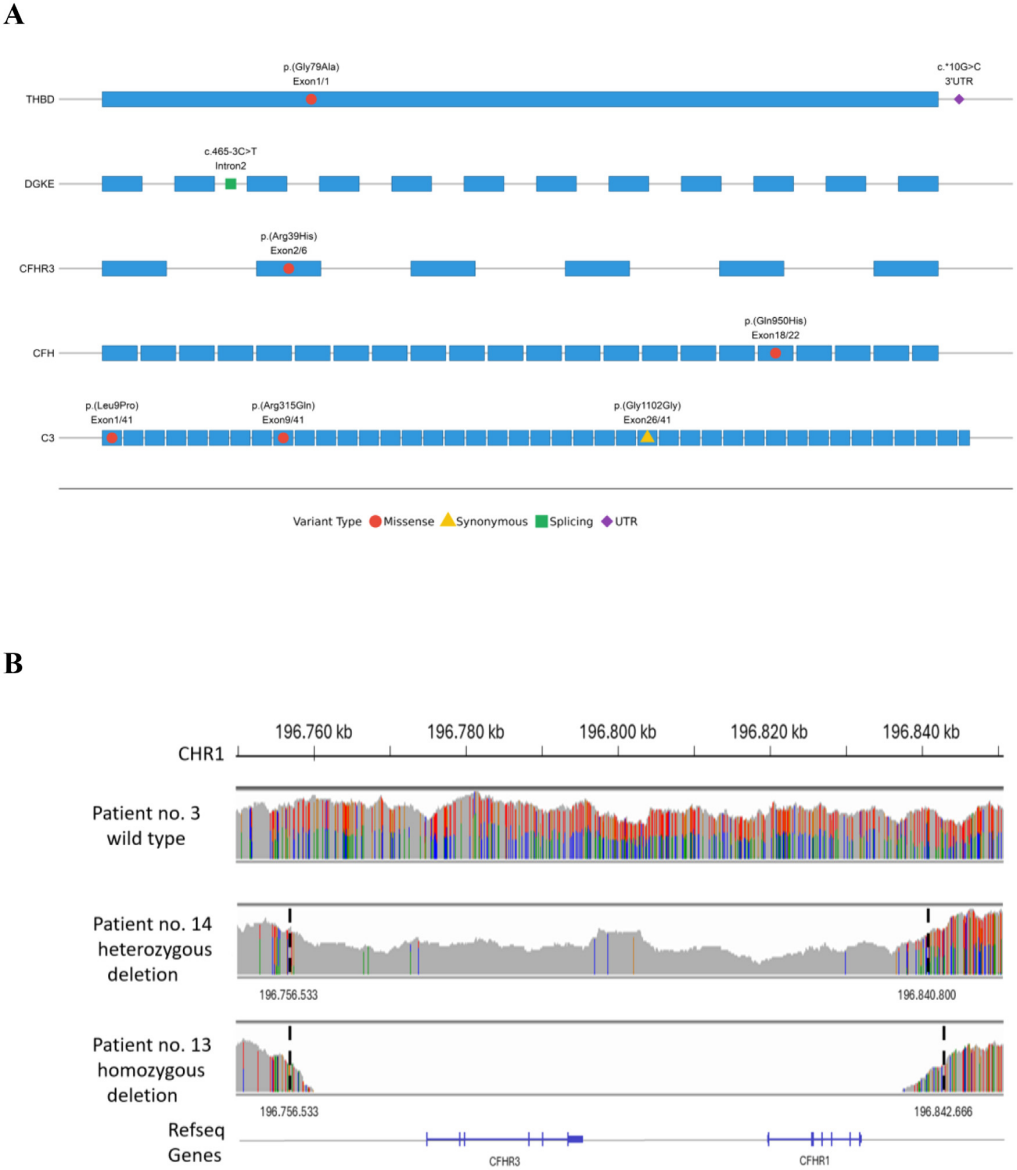
**A**. Variants in complement-related genes identified with next generation sequencing and confirmed with nanopore sequencing. The figure shows the single nucleotide variants (circles, squares, triangles and diamond) within the exons (blue rectangles) of the single complement- related genes (*C3*: NM_000064.4; *DGKE*: NM_003647.3, *THBD*: NM_000361.3; *CFH*: NM_000186.4; *CFHR3*: NM_021023.6. 3′UTR: 3′ untranslated region. **B.** Rapid genomic analysis in three patients with transplant-associated thrombotic microangiopathy (TA-TMA). The figure shows read coverage data across the genomic region encompassing the *CFHR3* and *CFHR1* genes for three different samples from patient no 3 (wild type), patient no 14 (heterozygous deletion) and patient no 13 (homozygous deletion). The dashed vertical lines indicate the breakpoints of the structural variants spanning the two *CFHR* genes, as automatically calculated by the CNVkit tool.

Table 6 shows complement genetic findings of the ten patients with TA-TMA and the five patients with <4 EAABMT criteria. All complement-related genetic variants identified in the 15 patients via targeted NGS were rare (MAF <1%) and classified as variants of uncertain significance (VUS). Seven variants were found in the group of ten TA-TMA patients (two patients had two variants and three patients had one variant) whereas only one variant was found in the group of five patients with <4 EAABMT criteria (Table 6 & Figure 2A). All variants were in the heterozygous state. The *CFHR3*-*CFHR1* deletion, identified by MLPA (Table 6), was present in four of ten patients with TA-TMA (one as homozygous and 3three as heterozygous) and in two of the five patients with <4 EAABMT criteria (all heterozygous). The slight difference in allele frequency (25% in patients with TA-TMA versus 20% in patients with <4 EAABMT criteria) did not reach statistical significance.

**Table 6:** Complement genetic data of 15 patients with suspected transplant-associated thrombotic microangiopathy (TA-TMA). Nanopore sequencing confirmed targeted next generation sequencing using the EPI2ME pipeline, whereas, to confirm MLPA deletions it was necessary to use the newly developed customized pipeline based on CNVKit.

Patients with confirmed diagnosis of transplant-associated thrombotic microangiopathy (TA-TMA) (EAABMT criteria ≥4) are highlighted in grey.

The 15 samples were sequenced with nanopore technology (Figure 2B) and analyzed by combining the EPI2ME and CNVKit pipelines. A custom in-house quality control tool was applied to ensure accurate variant calling within the complex *CFHR1*–*CFHR5* region. This analysis yielded a mean N50 of approximately 8400, a mean depth of 37×, and a mean alignment quality of 59.6. A drop in alignment quality was observed in the *CFHR1*–*CFHR3* region (39.4). The EPI2ME wf-human-variation workflow confirmed the results obtained with targeted NGS (Table 6) but failed to identify deletions detected by MLPA. In contrast, the CNVKit analysis focused on the *CFHR3*–*CFHR1* region, identified aberrations matching the MLPA location and zygosity for all six carrier samples. In the remaining samples, the tool produced no false positive calls, achieving 100% sensitivity and specificity for the analysis. Reproducibility tests performed on replicas of the same samples gave fully concordant results.

## Discussion

TA-TMA is a severe and often fatal complication following allo-HSCT; its diagnosis remains difficult due to the lack of universally accepted criteria and the overlapping of other post-transplant complications. Adult patients who met the diagnostic thresholds for TA-TMA in a single-center cohort of allo-HSCT recipients were identified according to the recently endorsed consensus criteria of major international transplantation societies [2]. Importantly, these criteria, while adapted from pediatric populations, have not been extensively validated in adults. The findings of this study support their applicability in this setting, demonstrating that patients meeting ≥4 EAABMT criteria had a higher case-fatality rate with a 1-year non-relapse mortality of 60% versus 20% in patients meeting <4 EAABMT criteria. In this study, the frequency of this life-threatening complication was about 5%; indeed ten out of 195 patients undergoing allo-HSCT had confirmed TA-TMA, consistent with the reports from Epperla et al. (3%) [30] and the more recent publication of Acosta-Medina et al. (6.3%) [31]. In this cohort, the presence of GvHD may have contributed to the development of TA-TMA via endothelial damage [32,33]. The clinical course of TA-TMA in this study further highlights its high morbidity and mortality rates. When comparing the ten patients with TA-TMA to the 185 without the complication, those who developed TA-TMA had a five-fold increased risk of death. Despite therapeutic interventions (including the discontinuation of calcineurin inhibitors in six patients (with clinical improvement in four) and eculizumab administration in one) outcomes remained poor, with a 1-year non-relapse mortality (NRM) of 60%. This rate is consistent with those reported in recent studies [31, 32, 33]. These observations underscore the need for earlier diagnosis and potentially more aggressive or targeted therapies. To facilitate timely intervention, this study introduces rapid nanopore sequencing for TA-TMA patients as a faster alternative to conventional NGS and MLPA techniques. Nanopore sequencing, with its capacity for real-time long-read analysis, enabled rapid detection and confirmation of structural variants in complement-related genes. Two different pipelines were used as the *CFHR3*-*CFHR1* deletion is in a high homology DNA region: the conventional pipeline and a customized one. This dual-pipeline strategy, including external validation with an independent bioinformatics strategy, provides strong technical support for the robustness of nanopore-based genomic profiling in this context. To the best of our knowledge, this is the first report to evaluate the utility of nanopore sequencing and rapid genomic analysis in the diagnostic workup of adult TA-TMA patients, thereby representing a significant advancement in the field. Compared with conventional approaches, nanopore technology holds promise for faster turnaround times and broader detection capabilities, which are critical for early interventions in TA-TMA. Seven rare genetic variants in complement-related genes were identified among these TA-TMA patients. All were classified as variants of uncertain significance (VUS) and have not been previously associated with thrombotic microangiopathies. However, in the post-allo-HSCT setting, these variants may confer increased susceptibility to TA-TMA following endothelial stressors, such as chemotherapy, calcineurin inhibitors, or GvHD, as proposed by the 'multiple-hit' model by Jodele et al. [15]. Most circulating complement is liver-derived (recipient-origin), meaning donor hematopoietic variants may have limited systemic impact. However, donor-derived immune cells produce complement locally; this local activation is likely a key driver of thrombotic microangiopathy in the microvasculature. This potential genetic susceptibility requires functional validation through donor–recipient comparisons in larger cohorts; at present, these findings remain hypothesis-generating. In the TA-TMA patients of this cohort, susceptibility to complement activation is supported by elevated sC5b-9 levels (a marker of terminal complement activation) that is considered a helpful tool in the diagnosis and disease monitoring in this population [34]. Indeed, complement inhibition therapy is proposed for patients exhibiting a clear complement activation according to the available biomarkers [34,35]. It could be argued that complement activation also occurs in conditions such as GvHD and infections, which frequently arise as complications in the post-transplantation period. However, the association of elevated sC5b-9 with ≥4 EAABMT criteria and the fact that GvHD and infections are present in patients without TA-TMA support the role of complement activation in TA-TMA rather than in GvHD or infection alone. In addition to validating clinical and biochemical indicators, this study demonstrates that donor-derived variants in complement-related genes are detectable in the peripheral blood of TA-TMA patients once full donor chimerism is achieved. These findings extend previous data regarding the potential role of donor genetic abnormalities in the pathogenesis of TA-TMA [17]. The utility of rapid complement profiling may extend to other post-transplant endothelial syndromes like SOS/VOD. Identifying the unique genetic drivers of these conditions is crucial, as their underlying involvement in the coagulation and complement systems differs from the specific pathways seen in TA-TMA [36].

This study has some limitations. The relatively small number of patients and the observational nature of the study limit the generalizability of the findings but prompted us to evaluate the genetic and phenotypic complement alterations in a large cohort of donors and recipients of allo-HSCT for a future study. The EAABMT consensus criteria represent an important advance in the diagnosis of TA-TMA, however these criteria require further adult validation. Additionally, the VUS classification of many genetic variants highlights the need for functional validation studies and larger multicenter genomic datasets to refine genotype-phenotype correlations in TA-TMA. In this study, genetic testing was performed after patients had already been treated, but the fact that rapid genomic analysis provides results comparable to time-consuming conventional tests suggests its potential in guiding timely therapeutic decisions.

In conclusion, the findings of this study support the diagnostic utility of EAABMT-based clinical criteria in adults with suspected TA-TMA and add novel evidence on the possibility of rapidly discovering complement genetic abnormalities by genomic analysis with nanopore sequencing. This may facilitate timely interventions. The role of complement genetic abnormalities in TA-TMA pathophysiology is further supported by elevated levels of sC5b-9. Future studies should aim to validate these findings in larger cohorts and investigate the integration of rapid genomic analysis to support real-time clinical decision-making in TA-TMA patients, as well as to potentially screen donors whose cells may carry genetic alterations

## Authorship

J.M., S.B. and M.C. designed the study; M.C. wrote the manuscript; J.M., S.B. B.S, and G.A. were responsible for the clinical management of the patient; L.P., M.T.P., S.S., SG and A.C. were responsible for the laboratory tests; D.M., E.A., M.U. and A.T. analyzed the genetic data; F.P. and A.S. organized the research work; all authors contributed to the interpretation of the results, critically reviewed the manuscript, and approved the final version for submission.

## Funding

This work was supported by the Piano Nazionale di Ripresa e Resilienza (PNRR), project Malattie Croniche non Trasmissibili (MCnT) ad alto impatto sui sistemi sanitari e socioassistenziali, code PNRR-MAD-2022–12376816, and by the Italian Ministry of Health – Bando Ricerca Corrente. The Fondazione IRCCS Ca’ Granda Ospedale Maggiore Policlinico is member of the European Reference Network on Rare Haematological Diseases EuroBloodNet-Project ID No 101157011. ERN-EuroBloodNet is partly co-funded by the European Union within the framework of the Fourth EU Health Programme. The Department of Pathophysiology and Transplantation, University of Milan, is funded by the Italian Ministry of Education and Research (MUR): Dipartimenti di Eccellenza Program 2023 to 2027. The contribution of E.A., M.U. and A.T. was supported by the PNC-E3–2022–23,683,266-INNOVA, WP6, Italian Ministry of Health.

## Conflicts of interest

G.A. is a member of the scientific advisory board of the Global Atypical Hemolytic Uremic Syndrome Registry supported by Alexion Pharmaceuticals Inc, and he has received honoraria from Alnylam, Roche, Novartis, and Alexion for his participation in scientific advisory boards or for giving lectures. F.P. has received honoraria for her participation in scientific advisory boards from Sobi, Sanofi, Roche, Biomarin, CSL Behring, Pfizer and for participating as a speaker in education meetings organized by Takeda, Spark and Sanofi. The remaining authors declare no conflicts of interest.

## Data Availability

The data that support the findings of this study are available from the corresponding author upon reasonable request.
